# Risk Factors for Mortality in Critically Ill Patients with Diabetes Admitted to the ICU: A Single-Center Retrospective Observational Study

**DOI:** 10.3390/jcm15062439

**Published:** 2026-03-23

**Authors:** Mădălina Diana Daina (Fehér), Codrin Dan Nicolae Ilea, Cosmin Mihai Vesa, Alina Cristiana Venter, Simona Daciana Birsan, Timea Claudia Ghitea, László Fehér, Cristian Marius Daina

**Affiliations:** 1 Doctoral School of Biomedical Sciences, Faculty of Medicine and Pharmacy, University of Oradea, 1 December Sq., 410081 Oradea, Romania; 2 Department of Psycho-Neurosciences and Recovery, Faculty of Medicine and Pharmacy, University of Oradea, 1 December Sq., 410081 Oradea, Romania; simona2372@yahoo.com (S.D.B.);; 3 Department of Preclinical Disciplines, Faculty of Medicine and Pharmacy, University of Oradea, 1 December Sq., 410081 Oradea, Romania; v_cosmin_15@yahoo.com; 4 Department of Morphological Disciplines, Faculty of Medicine and Pharmacy, University of Oradea, 410073 Oradea, Romania; aventer@uoradea.ro; 5 Pharmacy Department, Faculty of Medicine and Pharmacy, University of Oradea, 1 December Sq., 410081 Oradea, Romania

**Keywords:** critical patient, diabetes mellitus, ICU ward, risk of death

## Abstract

**Background and Objectives**: Diabetes mellitus (DM) is a highly prevalent comorbidity among critically ill patients and may significantly influence intensive care unit (ICU) outcomes through metabolic, immune, and cardiovascular mechanisms. This study aimed to evaluate the impact of DM on clinical profile, comorbidities, complications, need for intensive support, and mortality in adult ICU patients. **Materials and Methods**: A retrospective observational study was conducted between January and December 2024 in a tertiary ICU, including 1344 adult patients. Among them, 435 (32.37%) had DM. Demographic data, admission diagnoses, laboratory parameters, comorbidities, complications, therapeutic interventions, and outcomes were analyzed. Comparative statistical analysis and multivariate logistic regression were performed to identify independent predictors of ICU mortality. **Results**: Patients with DM were significantly older than patients without diabetes mellitus (non-DM group) (69.62 ± 10.26 vs. 67.16 ± 14.26 years, *p* < 0.001) and more frequently female (57%, *p* = 0.0002). At admission, they presented higher glycemia (204.7 vs. 134.0 mg/dL, *p* < 0.00001), reduced glomerular filtration rate (47.2 vs. 59.5 mL/min/1.73 m^2^, *p* < 0.00001), and more pronounced lymphocytopenia (*p* = 0.025). Cardiovascular and renal comorbidities were significantly more prevalent in DM, including hypertension (76.3%), heart failure (32.4%), and chronic kidney disease (33.1%) (all *p* < 0.01). DM was associated with increased odds of sepsis (OR 1.56), acute kidney injury (OR 1.51), and obesity (OR 2.57). ICU mortality was significantly higher in patients with DM (54.9% vs. 46.3%, *p* = 0.004; RR 1.19). Independent predictors of death included mechanical ventilation (OR 36.48), inotropic therapy (OR 4.74), hemodialysis (OR 2.57), elevated lactate, neutrophilia, and reduced glomerular filtration rate (GFR). **Conclusions**: DM was associated with increased ICU mortality and a higher burden of cardio-renal comorbidities and complications; however, mortality in the multivariate model was primarily driven by markers of organ dysfunction and the need for advanced supportive therapies. Early risk stratification and individualized management strategies are essential to improve outcomes in critically ill patients with diabetes.

## 1. Introduction

Diabetes mellitus (DM) represents one of the most important global health burdens, with a continuously increasing prevalence [1]. Updated estimates from the International Diabetes Federation [2] indicate that worldwide, over 530 million people live with diabetes, with 1 in 9 adults (20–79 years) presenting with DM [2,3]. The increasing trend of this condition is determined by a complex of factors that include population aging, urbanization and sedentary lifestyle, an increase that is inevitably reflected in the use of emergency services and intensive care [4,5,6,7]. In parallel, it is recognized that DM predisposes to severe infections and complicated clinical evolutions, through mechanisms that include immune dysfunction, chronic inflammation, endothelial dysfunction and pro-thrombotic status [8,9]. Therefore, legislative measures, prevention, early identification of risk factors, continuous monitoring, and confirmation in the context of the COVID-19 pandemic are crucial for reducing mortality among diabetic patients [10,11,12,13].

In intensive care units (ICUs), hyperglycemia is common in both patients with known DM and those without a history of DM, as an expression of stress hyperglycemia [14,15,16,17,18]. Data from the specialized literature show that 30–45% of hospitalized patients (and comparable percentages in ICUs) have hyperglycemia (>180 mg/dL), and in some study groups the percentages are even higher [1,14,19,20,21]. Hyperglycemia on admission, wide glycemic variations and episodes of severe hypo or hyperglycemia are frequently associated with increased mortality, longer length of hospitalization and infectious complications [22,23,24,25,26,27,28,29,30].

In this context, glycemic care in the ICU remains a delicate balance between avoiding prolonged hyperglycemia (associated with immune dysfunction, susceptibility to infections, and endothelial damage), limiting glycemic variability, and preventing hypoglycemia—an event with a negative prognostic impact [1,31,32,33,34,35,36,37,38,39,40,41]. The American Diabetes Association (ADA) 2025 standards and critical guidelines (SCCM) support the use of protocols with explicit decision, IV insulin infusions, and frequent monitoring, adapted to the patient’s particularities (age, renal failure, corticosteroid therapy, nutritional support). These principles are essential to correctly interpret the relationship between glycemic phenotype and mortality in the ICU in patients with DM.

Despite the high prevalence of diabetes mellitus among critically ill patients, its prognostic significance in the intensive care unit remains debated. Some studies suggest that diabetes is associated with increased morbidity and mortality, while others report that outcomes are primarily determined by the severity of acute organ dysfunction rather than the presence of diabetes itself [42,43,44]. Moreover, most available evidence originates from large multicenter databases, while fewer studies have explored how diabetes interacts with comorbidities, complications, and organ support requirements in real-world ICU populations at the regional level.

In this context, the present study aimed to characterize the clinical profile, comorbidity burden, complications, and outcomes of critically ill patients with diabetes admitted to a tertiary intensive care unit and to compare these parameters with those observed in patients without diabetes. We hypothesized that diabetes is associated with a distinct clinical and metabolic profile and may contribute to increased vulnerability during critical illness, even though mortality may ultimately be driven by the severity of acute organ dysfunction and the need for advanced supportive therapies.

## 2. Materials and Methods

### 2.1. Study Population

A retrospective observational study was conducted in the Intensive Care Unit (ICU) of the Bihor County Emergency Clinical Hospital, a tertiary referral center serving the population of Bihor County in north-western Romania, with an estimated catchment population of approximately 600,000 inhabitants.

**Figure 1:**
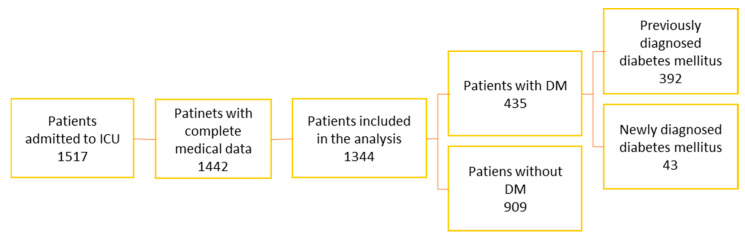
Study group selection.

During the analyzed period, 1517 patients were admitted to the ICU, representing all ICU admissions between 1 January and 31 December 2024 (patients without surgical intervention and patients with surgical intervention—postoperative) or transferred from other hospitals were admitted to the ICU. Patients with incomplete medical records (n = 75) and pediatric patients aged 0–17 years (n = 98) were excluded. After applying these exclusion criteria, 1344 adult patients were included in the final analysis (Figure 1).

Diabetes mellitus status was determined based on previous medical documentation (verified by the presence of patients in the County Diabetes Registry) and ongoing antidiabetic treatment (oral or insulin). The diagnosis of diabetes mellitus was established according to American Diabetes Association (ADA) criteria, including fasting plasma glucose ≥126 mg/dL, HbA1c ≥ 6.5%, or documented previous diagnosis of diabetes. When glycemic values ≥126 mg/dL were used as a diagnostic criterion, measurements were confirmed on two determinations performed under fasting conditions whenever clinical circumstances allowed. Patients without previous diagnosis, but with admission glycemia > 200 mg/dL and HbA1c ≥ 6.5%, were classified as “previously undiagnosed diabetes mellitus”. Patient classification was independently assessed by two physicians. Any discrepancies were resolved through discussion and consensus.

### 2.2. Methods and Collected Variables

For each adult patient, the following information was collected: demographic data (age, sex, place of residence—urban or rural), main cause of admission (acute and infectious diseases, respiratory and cardiovascular diseases, surgical interventions and other pathologies), relevant biological parameters (glycemia upon admission, blood count, serum creatinine, glomerular filtration rate, systolic and diastolic blood pressure, etc.), associated comorbidities (hypertension, ischemic coronary artery disease, heart failure, chronic renal failure, obesity, chronic liver disease, tuberculosis, chronic alcoholism (as documented in medical history), neoplasia and dyslipidemia, clinical evolution and interventions performed (development of sepsis or multiple organ failure, need for mechanical ventilation, use of inotropic agents, hemodialysis, insulin administration) and total duration of hospitalization in the ICU. Other forms of substance abuse were not consistently recorded in the medical records and were therefore not analyzed.

Acute respiratory failure was defined as the need for invasive mechanical ventilation or arterial oxygen tension (PaO_2_) <60 mmHg with respiratory distress.

Coma was defined as a Glasgow Coma Scale score ≤8.

Lactic acidosis was defined as serum lactate > 2 mmol/L associated with metabolic acidosis.

The data obtained were analyzed comparatively between groups of patients with DM and non-DM, in order to obtain statistical significance. Missing data were encountered for continuous variables as follows: glycemia—12 missing values, lactate—86 missing values, glomerular filtration rate (GFR)—59 missing values and pH—85 missing values. Missing data were present for several continuous variables (glycemia, lactate, GFR, and pH). Given the retrospective nature of the dataset and the relatively limited proportion of missing values, they were handled using simple mean imputation, applied to preserve the overall sample size in the regression analysis. However, this approach may underestimate variability for highly dispersed variables and should therefore be interpreted with caution. Future prospective studies could apply more advanced approaches such as multiple imputation. Confounding variables were selected based on the clinical relevance documented in the literature and preliminary significant correlations (*p* < 0.05) identified in the univariate analysis, being subsequently grouped into eight major pathophysiological categories: demographic and general, cardiovascular, respiratory and infectious, metabolic and endocrine, neurological, renal, hepatic/gastrointestinal and oncological/nutritional (Table A1). Demographic factors, pre-existing pathologies before admission to ICU, relevant biological parameters and therapeutic interventions were included in the multivariate logistic regression model. The backward elimination method was used to identify independent predictors of mortality.

### 2.3. Statistical Analysis

To verify the statistical hypotheses regarding categorical variables, Pearson’s chi-squared test (χ^2^ test) and Fisher’s test were used. To test continuous variables, the T-student test was applied. The statistical significance threshold was 0.05. For the regression model, a generalized linear regression using the backward elimination method was used. We started from the model with all variables included, eliminating them one by one, depending on the *p*-value. The significance threshold at which variables were considered significant for our model was 0.05. Normality of continuous variables was assessed using the Shapiro–Wilk test and inspection of distribution plots. Variables with approximately normal distribution were expressed as mean ± standard deviation and compared using Student’s *t*-test. To verify the regression model, R^2^ for the logistic regression developed by Nagelkerke, the Brier score, the Hosmer–Lemeshow calibration test and Harrell’s C statistic/Area under the ROC curve were calculated. Data analysis and application of statistical tests were done using the R program version 4.3.1.

### 2.4. Ethical Considerations

The study was conducted in accordance with the Declaration of Helsinki, with the approval of the Ethics Committee of the Bihor County Emergency Clinical Hospital no. 36976/30.1.2026 and the Opinion of the Ethics Council of the Bihor County Emergency Clinical Hospital no. 36746/30.1.2026, the data being anonymized to protect the confidentiality of the patients. Informed consent was waived due to the retrospective design and anonymized data collection.

## 3. Results

### 3.1. Demographic Characteristics and Prevalence of Diabetes Mellitus

**Figure 2:**
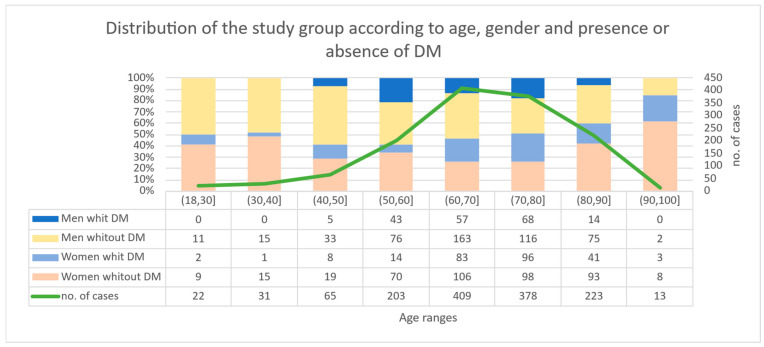
Distribution of the study group according to age, gender and presence or absence of DM.

The age of hospitalized patients (n = 1344 adult patients) ranged from 19 to 96 years, with a mean age of 67.95 ± 13.14 years and a median of 70 years. The largest proportion of hospitalized patients was between 60–79 years (58.56% of the total), with a peak in the 60–69 age range (409 patients, 30.4%). Age extremes were poorly represented: 22 patients 18–29 years (1.64%) and 13 patients 90–96 years (0.97%). By age group, women predominated in the 70–79 age group (194 women, 29.12%) and men in the 60–69 age group (220 men, 32.44%). DM was present in 435 patients (32.37%), and 909 patients (67.63%) had no diagnosed DM. DM prevalence increased with age, reaching maximum between 70–79 years (43.39%), with increased values also at 60–69 years (34.23%). Under 40 years DM was rare (3.2–9.1%), and in the intervals 40–49 and 50–59 years the proportion increased to 20.0% and 28.08%, respectively (Figure 2).

Women had a significantly higher mean age: 69.39 ± 13.29 years vs. 66.54 ± 12.86 years in men (*p* < 0.0001, Student’s *t* test). Patients with DM had a significantly higher average age than those without DM: 69.62 ± 10.26 years vs. 67.16 ± 14.26 years (*p* < 0.001). The median age was 71 years in patients with DM vs. 69 years in those without DM (Table 1).

### 3.2. Reason for Admission to ICU

In the DM group, 73.8% of patients were admitted for medical conditions and 26.2% for surgical indications. In contrast, among patients without DM, 64.4% were admitted for medical pathologies and 35.6% for surgical causes. The difference was statistically significant (*p* = 0.0007), indicating that diabetic patients were more frequently hospitalized for acute medical conditions rather than surgical procedures.

Analysis of admission diagnoses according to ICD-10 categories further clarified this distribution. The most prevalent diagnostic chapters in the overall cohort were respiratory diseases (29%), circulatory diseases (20.5%), neoplasms (17.2%), and digestive diseases (10.9%). Among patients with DM, respiratory diseases represented 36% of admissions, circulatory diseases 33.7%, and digestive diseases 32.2%. Endocrine and metabolic disorders showed the highest proportional association with DM (52.9%), reflecting mainly acute metabolic decompensations such as diabetic ketoacidosis (DKA) or hyperosmolar hyperglycemic state (HHS), as well as exacerbations of chronic metabolic conditions.

Sepsis and severe infections were more frequent in patients with DM (22.1% vs. 15.4%, *p* = 0.003), supporting the concept of increased infectious vulnerability in this population. Additionally, genitourinary diseases were more commonly associated with DM (40.8%), while trauma-related and injury diagnoses were significantly less prevalent in diabetic patients (14.1%), consistent with the lower proportion of surgical and trauma admissions.

Overall, diabetic patients exhibited a predominantly medical and infection-driven admission profile, characterized by respiratory, cardiovascular, renal, and metabolic conditions. In contrast, non-diabetic patients more frequently presented with acute surgical or trauma-related pathologies. These findings suggest that DM contributes to a systemic vulnerability that predisposes patients to severe medical conditions requiring intensive care rather than isolated surgical events (Table 2).

### 3.3. Biological Status upon Admission

The analysis of admission laboratory parameters revealed significant metabolic and renal differences between patients with and without DM, while inflammatory markers were largely comparable between groups.

As expected, mean admission glycemia was significantly higher in patients with DM (204.74 ± 134.67 mg/dL) compared to non-diabetic patients (134.04 ± 72.31 mg/dL, *p* < 0.00001). This reflects both pre-existing glycemic dysregulation and stress-induced hyperglycemia in the critical setting.

Renal function was significantly impaired in the DM group. The mean glomerular filtration rate (GFR) was markedly lower in diabetic patients (47.19 ± 31.52 mL/min/1.73 m^2^) compared to those without DM (59.48 ± 35.43 mL/min/1.73 m^2^, *p* < 0.00001). Although serum creatinine values did not differ significantly, the reduced GFR suggests a higher burden of underlying chronic kidney disease or increased susceptibility to acute renal dysfunction among patients with DM.

Regarding hematological parameters, total leukocyte and neutrophil counts did not differ significantly between groups (*p* > 0.05), indicating a comparable acute inflammatory response at admission. However, lymphocyte counts were significantly lower in patients with DM (1.49 ± 3.82 × 10^3^/µL vs. 4.58 ± 4.13 × 10^3^/µL, *p* = 0.025), suggesting a degree of immune compromise in the diabetic population.

No statistically significant differences were observed in platelet count, lactate levels, blood pressure values (SBP and DBP), or arterial pH at admission, although diabetic patients showed a non-significant trend toward lower pH values.

Overall, patients with DM presented at ICU admission with a more pronounced metabolic imbalance and impaired renal function, accompanied by relative lymphocytopenia, indicating increased metabolic and immunological vulnerability at the onset of critical illness (Table 3).

### 3.4. Comorbidities

Comorbidities were highly prevalent in the overall cohort, affecting 88.17% of patients. However, their distribution differed significantly between groups. Patients with DM presented a higher overall burden of comorbidities compared to non-diabetic patients (92.64% vs. 86.03%, *p* = 0.0006).

Cardiovascular diseases were markedly more frequent in the DM group. Arterial hypertension was the most prevalent comorbidity, affecting 76.32% of diabetic patients compared to 56.33% of non-diabetic patients (*p* < 0.00001). Similarly, heart failure was significantly more common in patients with DM (32.41% vs. 22.33%, *p* = 0.00009), as was chronic coronary artery disease (47.59% vs. 39.05%, *p* = 0.003). Peripheral arterial disease was also more frequent among diabetic patients (5.52% vs. 3.08%, *p* = 0.044), supporting the association between DM and macrovascular pathology.

Renal and metabolic comorbidities showed the most pronounced differences. Chronic kidney disease was present in 33.10% of patients with DM compared to 14.41% of those without DM (*p* < 0.00001), representing more than a twofold difference. Obesity was also significantly more prevalent in the diabetic group (25.29% vs. 11.66%, *p* < 0.00001), reflecting the metabolic burden frequently associated with type 2 diabetes.

In contrast, some conditions were more common in non-diabetic patients, including chronic alcoholism (10.56% vs. 5.75%, *p* = 0.005) and pulmonary tuberculosis (1.43% vs. 0.23%, *p* = 0.045). The prevalence of chronic obstructive pulmonary disease, dementia, cirrhosis, dyslipidemia, and most valvular heart diseases did not differ significantly between groups.

Overall, diabetic patients admitted to the ICU exhibited a significantly higher cardio-renal and metabolic comorbidity burden, which likely contributes to their increased clinical vulnerability and adverse outcomes (Table 4).

### 3.5. Diabetes Mellitus, Risk Factor

To evaluate whether DM was associated with specific acute or chronic conditions at ICU admission, odds ratios (OR) and relative risks (RR) were calculated for pathologies showing statistically significant differences between groups (*p* < 0.05).

DM was significantly associated with an increased risk of severe infectious conditions. Patients with DM had higher odds of developing sepsis (OR 1.56; RR 1.43; *p* = 0.003) and other infectious complications (OR 1.44; RR 1.36; *p* = 0.03), supporting the concept of increased infectious susceptibility in this population.

Cardiovascular conditions were also more strongly associated with DM. Hypertension showed a particularly strong association (OR 2.39; RR 1.36; *p* < 0.00001). Heart failure (OR 1.66; RR 1.45; *p* = 0.00009) and chronic ischemic heart disease (OR 1.39; RR 1.22; *p* = 0.003) were significantly more frequent among diabetic patients, indicating a pronounced macrovascular burden.

Renal and metabolic conditions demonstrated some of the highest risk estimates. Chronic kidney disease was strongly associated with DM (OR 2.52; RR 2.30; *p* < 0.00001), while obesity also showed a robust association (OR 2.57; RR 2.17; *p* < 0.00001). Acute kidney injury at admission was more common in patients with DM (OR 1.51; RR 1.33; *p* = 0.003). Additionally, lactic acidosis was modestly but significantly associated with DM (OR 1.41; RR 1.38; *p* = 0.04), reflecting increased metabolic instability in critically ill diabetic patients.

Overall, these findings indicate that DM acts as a systemic risk amplifier in the ICU setting, being strongly linked to infectious, cardiovascular, renal, and metabolic pathologies. The magnitude of the associations, particularly for chronic kidney disease, obesity, and hypertension, highlights the multidimensional burden carried by diabetic patients at the time of critical illness (Table 5).

### 3.6. Clinical Evolution

The clinical course during ICU hospitalization was evaluated in terms of complications, need for supportive interventions, length of stay, and mortality.

Complications occurred in 734 patients (54.61% of the total cohort). Their incidence was significantly higher in patients with DM compared to those without DM (60.0% vs. 52.04%, *p* = 0.007), indicating a more unstable clinical evolution in the diabetic group. Major complications, particularly respiratory failure and sepsis, were more frequently observed in patients with DM.

Regarding supportive interventions, mechanical ventilation was required in approximately 71% of patients in both groups, without significant differences (*p* = 0.6), suggesting that the need for ventilatory support was primarily driven by the severity of the acute illness rather than diabetic status alone. Inotropic support was slightly more frequent in patients with DM (34.02% vs. 29.04%), although this difference did not reach statistical significance (*p* = 0.07).

In contrast, renal replacement therapy was significantly more common in diabetic patients (10.80% vs. 6.05%, *p* = 0.003), consistent with the higher prevalence of chronic kidney disease and acute kidney injury in this group. As expected, insulin therapy was administered far more frequently in patients with DM (71.72% vs. 4.84%, *p* < 0.00001), reflecting glycemic management in critical illness.

The mean length of ICU stay was slightly longer in patients with DM (7.99 vs. 7.54 days), but the difference was not statistically significant (*p* = 0.44).

ICU mortality was significantly higher among diabetic patients (54.94%) compared to non-diabetic patients (46.31%, *p* = 0.004), corresponding to a relative risk of death of 1.19. In contrast, mortality after transfer from the ICU to other hospital wards did not differ significantly between groups.

Overall, although DM did not significantly prolong ICU length of stay or increase the need for ventilatory support, it was associated with a higher rate of complications, greater need for renal replacement therapy, and significantly increased ICU mortality (Table 6).

### 3.7. The Risk of Death

To identify factors associated with ICU mortality, odds ratios (OR) were calculated for pre-existing conditions, acute complications, and intensive care interventions. Only statistically significant variables (*p* < 0.05) are reported.

Univariate analysis identified several pre-existing, acute, and critical care variables associated with ICU mortality (Table 7).

Among chronic conditions, chronic kidney disease, heart failure, atrial fibrillation, and prior stroke were significantly associated with increased risk of death.

Acute complications demonstrated stronger associations, particularly acute respiratory failure (OR 2.87), acute kidney injury (OR 2.72), lactic acidosis (OR 2.80), and coma (OR 2.36).

Severe critical events showed the highest mortality risk, especially septic shock (OR 3.75) and resuscitated cardiorespiratory arrest (OR 4.19).

The need for life-sustaining therapies was strongly correlated with mortality, with mechanical ventilation showing the highest association (OR 51.88), followed by inotropic support and hemodialysis.

#### 3.7.1. Pre-Existing Conditions

Among chronic comorbidities, chronic kidney disease (OR 2.75), heart failure (OR 1.60), atrial fibrillation (OR 1.75), and chronic ischemic heart disease (OR 1.29) were significantly associated with increased mortality in the overall cohort. In contrast, certain conditions such as dyslipidemia (OR 0.49) and neoplastic diseases (OR 0.50) were associated with lower observed mortality, possibly reflecting differences in admission profiles or competing risks.

In the subgroup analysis of patients with DM, chronic kidney disease (OR 1.65), heart failure (OR 1.56), atrial fibrillation (OR 1.63), and prior stroke (OR 1.73) remained significantly associated with death, suggesting that in diabetic patients, cardio-renal and neurological impairment play a major prognostic role.

#### 3.7.2. Acute Complications

Acute conditions developed during ICU stay were among the strongest predictors of mortality. Acute respiratory failure was significantly associated with death in the overall cohort (OR 2.87) and showed an even stronger association in patients with DM (OR 3.38). Acute kidney injury (OR 2.72), stroke (OR 1.66), coma (OR 2.36), pneumonia (OR 1.64), bronchopneumonia (OR 1.85), sepsis (OR 1.83), and lactic acidosis (OR 2.80) were all significantly linked to increased mortality.

Among the most severe complications, septic shock (OR 3.75), cardiogenic shock (OR 3.08), hepatorenal syndrome (OR 3.77), and resuscitated cardiorespiratory arrest (OR 4.19) were associated with the highest mortality risks. In patients with DM, resuscitated cardiorespiratory arrest showed a particularly elevated OR (7.22), indicating a disproportionate impact of terminal events in this subgroup.

Hypoglycemia, although infrequent, was associated with a markedly increased risk of death in the overall cohort (OR 5.78), highlighting the prognostic relevance of severe glycemic instability.

#### 3.7.3. Intensive Care Interventions

The need for life-sustaining therapies was strongly associated with mortality. Mechanical ventilation showed the highest association (OR 51.88 overall), followed by inotropic support (OR 9.23), hemodialysis (OR 4.42), and insulin therapy (OR 1.89). These variables likely reflect the severity of organ dysfunction rather than independent causal effects.

#### 3.7.4. Multivariate Analysis

Multivariate logistic regression identified mechanical ventilation (OR 36.48), inotropic therapy (OR 4.74), hemodialysis (OR 2.57), insulin therapy (OR 1.46), reduced GFR, elevated lactate, and neutrophilia as independent predictors of ICU mortality. The model demonstrated good discrimination (AUC > 85%) and calibration.

Overall, while DM modestly increased mortality risk, the principal determinants of death were acute multiorgan dysfunction and the need for advanced supportive therapies. However, in diabetic patients, the impact of severe complications appeared amplified, suggesting increased vulnerability to critical decompensation (Table 8).

**Figure 3:**
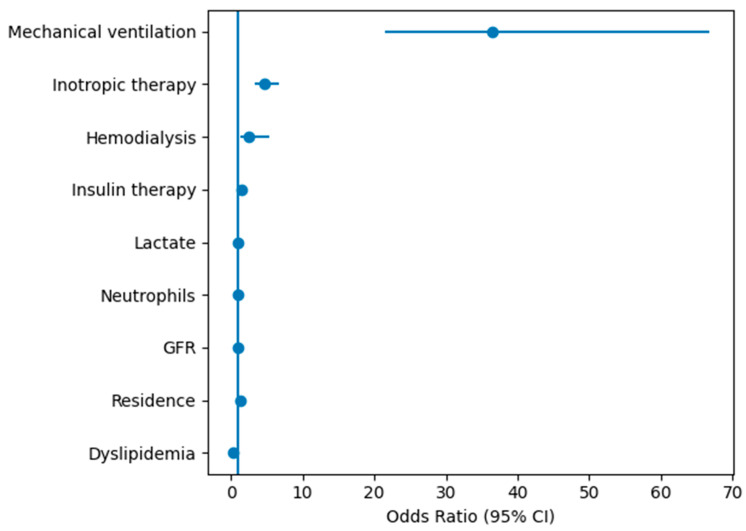
Forest plot illustrating independent predictors of ICU mortality identified by multivariate logistic regression analysis. Points represent odds ratios (OR) and horizontal lines indicate 95% confidence intervals. The vertical reference line corresponds to OR = 1.

Multivariate logistic regression identified mechanical ventilation, inotropic therapy, hemodialysis, insulin therapy, lactate, neutrophils, GFR, residence and dyslipidemia as independent predictors of ICU mortality (Table 8). The independent predictors identified by multivariate logistic regression are graphically illustrated in Figure 3.

## 4. Discussion

The present study evaluated the clinical profile, complications, and outcomes of critically ill patients with diabetes mellitus admitted to a tertiary intensive care unit. The main finding is that although diabetes was associated with a higher burden of cardiometabolic comorbidities, infectious complications, and increased crude ICU mortality, the multivariate analysis indicates that mortality was primarily driven by markers of acute organ dysfunction and the need for advanced supportive therapies. These findings suggest that diabetes represents an important vulnerability factor in critically ill patients but does not independently determine ICU outcomes once severe physiological derangements occur [45,46,47,48].

This retrospective study highlights the substantial burden of DM among critically ill adults, who were predominantly female (57%), while men predominated in the non-DM group. This imbalance may reflect demographic differences in longevity and the higher post-menopausal DM risk reported in multicenter analyses [49]. Importantly, advanced age and female sex have been associated with worse ICU outcomes in diabetic patients, supporting the inclusion of these parameters in risk stratification approaches and severity assessment frameworks [50].

A key finding of this study is the distinct admission profile of diabetic patients, who were significantly more often admitted for medical rather than surgical indications (73.8% vs. 64.4%, *p* = 0.0007). This pattern is consistent with previous evidence showing that severe infections, respiratory failure, and acute metabolic derangements are dominant drivers of ICU admission in patients with DM [31,51,52]. Respiratory diseases were the most frequent admission category in the DM group, followed by circulatory and digestive diseases, a distribution coherent with studies reporting increased risks of pneumonia, acute respiratory failure, and sepsis in diabetic patients [53]. The higher sepsis prevalence in DM (22.1% vs. 15.4%, *p* = 0.003) further supports the concept of diabetes-related immune vulnerability and infection severity [54,55]. Additionally, atrial fibrillation was more frequent in DM (31.3% vs. 24.8%, *p* = 0.01), consistent with literature linking chronic hyperglycemia and hypertension to atrial remodeling and arrhythmogenic substrate development [56,57]. Acute kidney injury and metabolic acidosis were also more common in diabetic patients, suggesting early and clinically relevant renal–metabolic instability in the context of critical illness [58,59]. Conversely, the lower prevalence of trauma-related and surgical admissions among diabetic patients suggests that DM primarily contributes to systemic, infection-driven, and metabolic admissions rather than acute traumatic indications. These findings collectively reinforce the need for early sepsis recognition, close renal monitoring, and careful glycemic management in critically ill diabetic patients, in accordance with current practice guidelines [3,28,60].

Our findings are consistent with several large observational studies evaluating the impact of diabetes in critically ill populations. Large ICU cohorts have reported diabetes prevalence between 20% and 40% among critically ill adults, with variable effects on mortality depending on case mix and baseline severity. For example, analyses of large ICU databases such as MIMIC and multicenter observational studies have suggested that diabetes is frequently associated with increased comorbidity burden and metabolic instability, while mortality risk is often primarily driven by acute organ dysfunction rather than diabetes status alone. These observations are consistent with our findings, where the strongest mortality predictors were markers of organ failure and the need for advanced organ support.

At ICU admission, diabetic patients displayed a more severe metabolic and renal profile. Mean glycemia was significantly higher in DM (204.7 mg/dL vs. 134.7 mg/dL, *p* < 0.00001), a finding consistent with the high prevalence of hyperglycemia in critical illness. Stress hyperglycemia has repeatedly been associated with increased mortality across ICU cohorts, independent of prior diabetic status [26,32]. However, the optimal glucose target remains a balance between avoiding prolonged hyperglycemia and preventing hypoglycemia, as overly strict control may be harmful; contemporary recommendations generally support a target range of 140–180 mg/dL in critically ill patients [35,36,37,38,39]. While leukocyte and neutrophil counts did not differ significantly, lymphocyte counts were lower in DM (*p* = 0.025), suggesting immune impairment and aligning with evidence linking lymphocytopenia to severe infection risk [48,61]. Renal vulnerability was further supported by significantly lower GFR in diabetic patients (*p* < 0.00001), even in the absence of creatinine differences, indicating either a higher burden of underlying chronic kidney disease or increased susceptibility to acute renal dysfunction—an association widely documented in ICU literature [62]. Trends toward lower pH and higher lactate, although not statistically significant, may still justify careful perfusion and metabolic assessment given their established relevance in critical illness severity [8,9].

The comorbidity burden was markedly higher in diabetic patients (92.6% vs. 86.0%, *p* = 0.0006), dominated by cardiovascular, renal, and metabolic conditions. Hypertension, heart failure, and chronic coronary artery disease were significantly more prevalent in DM, reflecting the interrelationship between hyperglycemia, endothelial dysfunction, and accelerated atherosclerosis [63]. The higher frequency of peripheral arterial disease supports the contribution of macrovascular diabetic complications to ICU vulnerability [64]. Chronic kidney disease was substantially more frequent in DM, consistent with diabetes being a leading cause of chronic kidney disease worldwide and with evidence indicating a high renal comorbidity burden in critically ill diabetic populations [65]. Obesity, also significantly more prevalent in DM, represents a key metabolic syndrome component and may further worsen insulin resistance and contribute to complications in hospitalized patients [66].

Another important question raised in the literature is whether diabetes modifies the host response to severe infections such as sepsis or septic shock. Chronic hyperglycemia has been associated with immune dysfunction, impaired neutrophil activity, endothelial dysfunction, and a pro-inflammatory state, which may increase susceptibility to infection. However, evidence regarding the impact of diabetes on sepsis outcomes remains heterogeneous. Some studies suggest increased infection risk but not necessarily higher mortality once severe sepsis develops. In our cohort, diabetes was associated with a higher prevalence of infectious complications, including sepsis, but mortality appeared to be primarily determined by the severity of acute organ dysfunction rather than diabetes status alone.

Beyond comorbidity burden, our OR/RR analysis indicates that DM functions as a systemic risk amplifier in the ICU. Diabetic patients had increased odds of sepsis (OR 1.56; RR 1.43) and infections (OR 1.44; RR 1.36), consistent with multicenter data describing higher sepsis incidence and infection severity among diabetic ICU patients [67,68]. Cardiovascular conditions—including hypertension and heart failure—also demonstrated significant associations, reflecting advanced cardiometabolic disease in this population and the effects of chronic glycemic injury on vascular stiffness and cardiac remodeling [63]. Furthermore, chronic kidney disease and the increased need for renal replacement therapy support the role of DM in renal vulnerability, through both chronic diabetic nephropathy and susceptibility to acute deterioration [65].

Clinically, diabetic patients had higher complication rates (60% vs. 52%, *p* = 0.007), consistent with reports showing increased multiorgan dysfunction risk among ICU patients with DM [32,69]. They required hemodialysis more frequently and insulin therapy at markedly higher rates, while mechanical ventilation and inotropic support showed no significant intergroup differences. This suggests that the presence of DM may not primarily drive initial cardiorespiratory support needs, but is strongly linked to renal–metabolic instability during critical illness. These findings are consistent with guideline-based approaches emphasizing avoidance of sustained hyperglycemia while minimizing hypoglycemia risk, without implying that DM alone should predict ventilatory requirements [60].

Despite only a marginal, non-significant difference in ICU length of stay, diabetic patients had significantly higher ICU mortality (54.9% vs. 46.3%, *p* = 0.004), corresponding to an increased relative risk of death (~1.19). This aligns with evidence indicating that DM affects mortality more consistently than hospitalization duration [26]. Mortality in the ward after ICU transfer did not differ, suggesting that the impact of DM is most pronounced during the acute critical phase.

Mortality in diabetic ICU patients appears to be driven by a heterogeneous and interacting set of factors, including acute illness severity (often summarized by scores such as APACHE II or SOFA) [70], infectious burden and sepsis severity, cardio-renal comorbidity load, admission glycemic phenotype (stress hyperglycemia vs. known DM), glycemic variability, and hypoglycemia [71,72]. The literature suggests that stress hyperglycemia in non-diabetic patients may carry a stronger mortality signal than hyperglycemia in those with established DM, potentially reflecting maladaptive stress responses in acute disease [71,72]. Moreover, sepsis survivors with pre-existing DM may experience more frequent long-term cardiovascular sequelae, emphasizing the need for integrated short- and longer-term risk evaluation [73,74,75].

Our univariate analysis identified acute respiratory failure, sepsis, bronchopneumonia, coma, lactic acidosis, septic shock, and cardiorespiratory arrest as key mortality-associated variables, consistent with the role of multiorgan dysfunction as the principal determinant of ICU death and with Surviving Sepsis Campaign guidance [60]. These findings are in line with previous work describing DM as a negative prognostic factor in intensive care [58,76]. In the diabetic subgroup, severe acute complications and the need for life-sustaining therapies showed particularly strong associations with death, suggesting that diabetic patients may experience disproportionate mortality impact once multiorgan decompensation occurs.

Importantly, the multivariate regression model demonstrated good discrimination (AUC > 85%), supporting the clinical relevance of the identified independent predictors, including mechanical ventilation, inotropic therapy, hemodialysis, insulin therapy, and key biological parameters (neutrophils, lactate, GFR), along with selected contextual variables such as residence and dyslipidemia status. These results suggest that combining organ support requirements with renal function and inflammatory/metabolic markers can aid early risk stratification and individualized management of critically ill patients.

### Strengths and Limitations

A major strength of this study is the comprehensive evaluation of demographic characteristics, admission patterns, comorbidities, complications, supportive interventions, and mortality predictors, providing clinically applicable insights for early identification of high-risk diabetic patients, including potentially undiagnosed cases.

Several limitations should be acknowledged. The retrospective observational design limits causal inference and introduces potential sources of bias. First, the study population included both medical and surgical ICU patients, which may lead to selection bias because these groups differ substantially in admission indications, baseline risk profiles, and mortality patterns. In our cohort, diabetic patients were more frequently admitted for acute medical conditions, while non-diabetic patients had a relatively higher proportion of surgical admissions. This structural imbalance may represent a potential confounding factor when interpreting differences in complications and mortality. Some rare conditions produced unstable estimates due to small sample sizes. Post-discharge outcomes (e.g., 30-day mortality) were not available. Additionally, the cohort included both medical and surgical ICU patients, two groups with distinct baseline risks and admission indications. Surgical patients—often admitted for postoperative surveillance—generally had lower disease severity and lower DM prevalence, whereas diabetic patients were predominantly admitted for acute medical conditions. This structural imbalance may partially contribute to the observed differences in complications and mortality and should be considered when interpreting the results. Although separate stratified analyses would be informative, subgroup sizes limited robust additional modeling.

Another important limitation is the absence of standardized ICU severity scores such as the Acute Physiology and Chronic Health Evaluation II (APACHE II) or the Sequential Organ Failure Assessment (SOFA). These scores are widely used to adjust for baseline illness severity in ICU outcome studies. Without such adjustment, it cannot be excluded that part of the observed mortality difference reflects higher baseline disease severity among patients with diabetes rather than the independent effect of diabetes itself.

## 5. Conclusions

This study demonstrates a high prevalence of diabetes mellitus among critically ill patients and an association with increased ICU mortality and complication rates. However, in the multivariate model, mortality was primarily determined by markers of organ dysfunction and the need for advanced supportive therapies rather than diabetes status alone. Patients with DM were older and more frequently female, and were more commonly admitted for medical conditions, particularly infectious and respiratory diseases. At ICU admission, diabetic patients displayed a distinct cardio–renal–metabolic vulnerability, characterized by a higher comorbidity burden, marked hyperglycemia, lymphocytopenia, and reduced renal function. The increased burden of comorbidities in patients with DM was associated with a higher risk of sepsis, acute kidney injury, and major complications, resulting in a greater need for supportive interventions, especially hemodialysis and insulin therapy. Although ICU length of stay did not differ significantly between groups, ICU mortality was significantly higher in diabetic patients.

Diabetes mellitus was associated with a higher burden of comorbidities, metabolic disturbances, and ICU complications, as well as increased crude mortality. However, multivariate analysis indicated that mortality was primarily driven by markers of organ dysfunction and the need for advanced supportive therapies. These findings suggest that diabetes contributes to vulnerability in critical illness but does not independently determine mortality once severe multiorgan dysfunction develops.

## Figures and Tables

**Table 1:** Demographic characteristics of patients admitted to the ICU according to diabetes status.

Age	Whole Group	Women	Men	*T*-Test, *p*-Value	Without DM	With DM	*T*-Test, *p*-Value
Min	19	19	19		19	21	*p* < 0.001
Max	96	96	93		96	94	
Mean	67.95	69.39	66.54	*p* < 0.0001	67.16	69.62	
Standard deviation	13.14	13.29	12.86		14.26	10.26	
Median	70	71	67.5		69	71	
Confidence interval CI 95%		68.38–70.40	65.57–67.51		68.38–70.40	68.65–70.58	

Patients with DM were predominantly women: 248 women (57.0%) vs. 187 men (43.0%)—significant difference (*p* = 0.0002) compared to the group without DM, where men predominated: 491 men (54.0%) vs. 418 women (46.0%).

**Table 2:** Reason for ICU Admission and Distribution of Main Diagnostic Categories According to Diabetes Status.

Category	Total n (%)	Without DM n (%)	With DM n (%)	*p*-Value
Reason for admission				
Medical	906 (67.41%)	585 (64.36%)	321 (73.79%)	0.0007
Surgical	438 (32.59%)	324 (35.64%)	114 (26.21%)	
Main ICD-10 chapters				
Respiratory (J00–J99)	390 (29.0%)	250 (64.1%)	140 (35.9%)	—
Circulatory (I00–I99)	276 (20.5%)	183 (66.3%)	93 (33.7%)	—
Neoplasms (C00–D48)	231 (17.2%)	175 (75.8%)	56 (24.2%)	—
Digestive (K00–K93)	146 (10.9%)	99 (67.8%)	47 (32.2%)	—
Endocrine/metabolic (E00–E90)	17 (1.3%)	8 (47.1%)	9 (52.9%)	—
Genitourinary (N00–N99)	71 (5.3%)	42 (59.2%)	29 (40.8%)	—
Injury/trauma (S00–T98)	85 (6.3%)	73 (85.9%)	12 (14.1%)	—

ICU—intensive care unit; DM—diabetes mellitus; ICD-10—International Classification of Diseases, 10th Revision; n—number of patients. Data are presented as absolute numbers and percentages. Statistical comparison between medical and surgical admissions was performed using the chi-square test.

**Table 3:** Admission Biological Parameters According to Diabetes Status.

Parameter	Without DM (n = 909) Mean ± SD	With DM (n = 435) Mean ± SD	*p*-Value
Glycemia (mg/dL)	134.04 ± 72.31	204.74 ± 134.67	<0.00001
Leukocytes (×10^3^/µL)	14.22 ± 19.17	13.47 ± 10.50	0.35
Neutrophils (×10^3^/µL)	10.28 ± 6.72	11.73 ± 25.09	0.24
Lymphocytes (×10^3^/µL)	4.58 ± 4.13	1.49 ± 3.82	0.025
Platelets (×10^3^/µL)	238.00 ± 143.35	252.65 ± 137.89	0.072
Lactate (mg/dL)	16.79 ± 21.13	18.93 ± 24.29	0.13
Creatinine (mg/dL)	2.02 ± 4.41	2.43 ± 8.20	0.45
GFR (mL/min/1.73 m^2^)	59.48 ± 35.43	47.19 ± 31.52	<0.00001
SBP (mmHg)	120.41 ± 27.88	123.24 ± 30.93	0.11
DBP (mmHg)	71.73 ± 17.59	71.86 ± 16.79	0.89
pH	7.35 ± 0.13	7.32 ± 0.34	0.067

DM—diabetes mellitus; SD—standard deviation; GFR—glomerular filtration rate; SBP—systolic blood pressure; DBP—diastolic blood pressure; n—number of patients. Data are expressed as mean ± SD. Continuous variables were compared using Student’s *t*-test. Statistical significance was defined as *p* < 0.05.

**Table 4:** Major Comorbidities According to Diabetes Status.

Comorbidity	Without DM (n = 909) n (%)	With DM (n = 435) n (%)	*p*-Value
≥1 Comorbidity	782 (86.03%)	403 (92.64%)	0.0006
Hypertension	512 (56.33%)	332 (76.32%)	<0.00001
Chronic coronary artery disease	355 (39.05%)	207 (47.59%)	0.003
Heart failure	203 (22.33%)	141 (32.41%)	0.00009
Chronic kidney disease	131 (14.41%)	144 (33.10%)	<0.00001
Obesity	106 (11.66%)	110 (25.29%)	<0.00001
Peripheral arterial disease	28 (3.08%)	24 (5.52%)	0.044
Chronic alcoholism	96 (10.56%)	25 (5.75%)	0.005
Pulmonary tuberculosis	13 (1.43%)	1 (0.23%)	0.045

DM—diabetes mellitus; n—number of patients. Data are presented as absolute numbers and percentages. Comparisons between groups were performed using the chi-square test (or Fisher’s exact test where appropriate). Statistical significance was defined as *p* < 0.05.

**Table 5:** Conditions Significantly Associated with Diabetes Mellitus (OR and RR).

Category	Condition	OR	RR	*p*-Value
Infectious conditions	Sepsis	1.56	1.43	0.003
	Other infections	1.44	1.36	0.03
Cardiovascular conditions	Hypertension	2.39	1.36	<0.00001
	Heart failure	1.66	1.45	0.00009
	Chronic ischemic heart disease	1.39	1.22	0.003
	Atrial fibrillation	1.39	1.26	0.01
Renal and metabolic conditions	Chronic kidney disease	2.52	2.30	<0.00001
	Acute kidney injury	1.51	1.33	0.003
	Obesity	2.57	2.17	<0.00001
	Lactic acidosis	1.41	1.38	0.04

OR—odds ratio; RR—relative risk; Odds ratios and relative risks were calculated to evaluate the association between diabetes mellitus and the listed conditions. Comparisons were performed using the chi-square test. A *p*-value < 0.05 was considered statistically significant.

**Table 6:** Clinical Evolution According to Diabetes Status.

Outcome/Intervention	Without DM (n = 909) n (%)	With DM (n = 435) n (%)	*p*-Value
Any complication	473 (52.04%)	261 (60.00%)	0.007
Mechanical ventilation	642 (70.63%)	313 (71.95%)	0.60
Inotropic support	264 (29.04%)	148 (34.02%)	0.07
Hemodialysis	55 (6.05%)	47 (10.80%)	0.003
Insulin therapy	44 (4.84%)	312 (71.72%)	<0.00001
ICU mortality	421 (46.31%)	239 (54.94%)	0.004
Post-ICU ward mortality	23 (2.53%)	12 (2.76%)	0.90

DM—diabetes mellitus; ICU—intensive care unit; n—number of patients. Data are presented as absolute numbers and percentages. Comparisons between groups were performed using the chi-square test (or Fisher’s exact test where appropriate). Statistical significance was defined as *p* < 0.05.

**Table 7:** Major Factors Associated with ICU Mortality (Univariate Analysis).

Category	Variable	OR (95% CI)	*p*-Value
Pre-existing conditions	Chronic kidney disease	2.75 (2.13–3.56)	<0.05
	Heart failure	1.60 (1.25–2.06)	<0.05
	Atrial fibrillation	1.75 (1.30–2.35)	<0.05
	History of stroke	1.48 (1.12–1.96)	<0.05
Acute complications	Acute respiratory failure	2.90 (2.29–3.67)	<0.05
	Acute kidney injury	2.75 (2.13–3.56)	<0.05
	Sepsis	1.83 (1.37–2.45)	<0.05
	Bronchopneumonia	1.86 (1.43–2.42)	<0.05
	Coma	2.40 (1.86–3.10)	<0.05
	Lactic acidosis	2.81 (2.03–3.90)	<0.05
Severe critical events	Septic shock	3.94 (2.54–6.09)	<0.05
	Cardiogenic shock	2.98 (1.38–6.44)	<0.05
	Resuscitated cardiorespiratory arrest	4.24 (2.73–6.59)	<0.05
Intensive care interventions	Mechanical ventilation	51.35 (30.11–87.57)	<0.05
	Inotropic support	9.34 (6.99–12.47)	<0.05
	Hemodialysis	4.56 (2.76–7.55)	<0.05
	Insulin therapy	1.91 (1.49–2.44)	<0.05

ICU—intensive care unit; OR—odds ratio; CI—confidence interval; ICU—intensive care unit. Univariate logistic regression analysis was performed to evaluate the association between each variable and ICU mortality. Results are expressed as odds ratios (OR) with 95% confidence intervals (CI). A *p*-value < 0.05 was considered statistically significant.

**Table 8:** Independent Predictors of ICU Mortality (Multivariate Logistic Regression).

Variable	OR	95% CI	*p*-Value
Mechanical ventilation	36.48	21.47–66.75	<0.001
Inotropic therapy	4.74	3.41–6.66	<0.001
Hemodialysis	2.57	1.29–5.42	0.010
Insulin therapy	1.46	1.04–2.05	0.028
Lactate	1.01	1.00–1.02	0.012
Neutrophils	1.03	1.01–1.05	0.017
GFR	0.99	0.99–1.00	<0.001
Residence	1.37	1.03–1.84	0.032
Dyslipidemia	0.29	0.16–0.53	<0.001

ICU—intensive care unit; OR—odds ratio; CI—confidence interval; GFR—glomerular filtration rate. Multivariate logistic regression analysis was performed to identify independent predictors of ICU mortality. Results are expressed as odds ratios (OR) with 95% confidence intervals (CI). Variables were selected using the backward elimination method. A *p*-value < 0.05 was considered statistically significant.

## Data Availability

The raw data supporting the conclusions of this article will be made available by the authors on request.

## Abbreviations

The following abbreviations are used in this manuscript:

DM	Diabetes mellitus
ICU	Intensive care unit
GFR	Glomerular filtration rate
AKI	Acute kidney injury
CKD	Chronic kidney disease
OR	Odds ratio
CI	Confidence interval

## Appendix A

**Table A1:** Grouping of confounding variables used in multivariate analysis, depending on the pathophysiological domain.

Category	Variables
Demographic and general data	age
	sex
	residence
	days in ICU
Cardiovascular and hemodynamic factors	chronic ischemic heart disease
	heart failure
	atrial fibrillation
	dilated cardiomyopathy
	acute coronary syndrome
	cardiogenic shock
	inotropic support
Respiratory and infectious factors	acute respiratory failure
	pneumonia
	bronchopneumonia
	pleurisy
	sepsis
	septic shock
	infections
	mechanical ventilation
	sleep apnea syndrome
Metabolic and endocrine factors	diabetes mellitus
	dyslipidemia
	hypoglycemia
	lactic acidosis
	complications
	insulin therapy
Neurological and neurovascular factors	coma
	stroke
	history of stroke
	seizures
	resuscitated cardiorespiratory arrest
Renal and urinary factors	acute kidney injury
	chronic kidney disease
	hepatorenal syndrome
	nephrolithiasis
	hemodialysis
Hepatic and gastrointestinal factors	cirrhosis
	vascular and parenchymal decompensated ethanol cirrhosis
	liver failure
	acute pancreatitis
	gastrointestinal bleeding
Oncological and nutritional factors	neoplastic diseases
	tumors
	hypoproteinemia
